# Remote Training of Functional Endoscopic Sinus Surgery With Advanced Manufactured 3D Sinus Models and a Telemedicine System

**DOI:** 10.3389/fsurg.2021.746837

**Published:** 2021-10-01

**Authors:** Masanobu Suzuki, Erich Vyskocil, Kazuhiro Ogi, Kotaro Matoba, Yuji Nakamaru, Akihiro Homma, Peter J. Wormald, Alkis J. Psaltis

**Affiliations:** ^1^Department of Surgery–Otorhinolaryngology Head and Neck Surgery, Central Adelaide Local Health Network and the University of Adelaide, Adelaide, SA, Australia; ^2^Department of Otolaryngology-Head and Neck Surgery, Faculty of Medicine and Graduate School of Medicine, Hokkaido University, Sapporo, Japan; ^3^Department of Forensic Medicine, Faculty of Medicine and Graduate School of Medicine, Hokkaido University, Sapporo, Japan

**Keywords:** surgical training, functional endoscopic sinus surgery (FESS), 3D printer, building block concept, frontal sinusotomy, social distancing regulation

## Abstract

**Objective:** Traditionally, cadaveric courses have been an important tool in surgical education for Functional Endoscopic Sinus Surgery (FESS). The recent COVID-19 pandemic, however, has had a significant global impact on such courses due to its travel restrictions, social distancing regulations, and infection risk. Here, we report the world-first remote (Functional Endoscopic Sinus Surgery) FESS training course between Japan and Australia, utilizing novel 3D-printed sinus models. We examined the feasibility and educational effect of the course conducted entirely remotely with encrypted telemedicine software.

**Methods:** Three otolaryngologists in Hokkaido, Japan, were trained to perform frontal sinus dissections on novel 3D sinus models of increasing difficulty, by two rhinologists located in Adelaide, South Australia. The advanced manufactured sinus models were 3D printed from the Computed tomography (CT) scans of patients with chronic rhinosinusitis. Using Zoom and the Quintree telemedicine platform, the surgeons in Adelaide first lectured the Japanese surgeons on the Building Block Concept for a three Dimensional understanding of the frontal recess. They in real time directly supervised the surgeons as they planned and then performed the frontal sinus dissections. The Japanese surgeons were asked to complete a questionnaire pertaining to their experience and the time taken to perform the frontal dissection was recorded. The course was streamed to over 200 otolaryngologists worldwide.

**Results:** All dissectors completed five frontal sinusotomies. The time to identify the frontal sinus drainage pathway (FSDP) significantly reduced from 1,292 ± 672 to 321 ± 267 s (*p* = 0.02), despite an increase in the difficulty of the frontal recess anatomy. Image analysis revealed the volume of FSDP was improved (2.36 ± 0.00 to 9.70 ± 1.49 ml, *p* = 0.014). Questionnaires showed the course's general benefit was 95.47 ± 5.13 in dissectors and 89.24 ± 15.75 in audiences.

**Conclusion:** The combination of telemedicine software, web-conferencing technology, standardized 3D sinus models, and expert supervision, provides excellent training outcomes for surgeons in circumstances when classical surgical workshops cannot be realized.

## Introduction

Functional Endoscopic Sinus Surgery (FESS) is the standard surgical procedure for chronic rhinosinusitis (1). To guarantee safe and consistent surgical outcomes of FESS, a clear and complete understanding of the three dimensional (3D) anatomy of paranasal sinuses is required (2). The frontal sinus is the most complicated and difficult of all sinuses and has a significant anatomical variation (2–4). As the frontal sinus is adjacent to critical structures such as the orbit and skull base, the risk of complications is higher compared to other sinuses. The Building Block Concept (BBC) has been proposed as an excellent tool for surgeons to preoperatively understand the anatomy of the frontal sinus (5). It allows the surgeon to identify each cell in the frontal recess as well as the frontal sinus drainage pathway (FSDP), thereby facilitating a structured surgical approach to this region.

Besides a clear understanding of the anatomy, surgical technique has to be taught, practiced and acquired to achieve consistent and safe outcomes. Along with literature, textbooks, and training in theater, surgical training courses are the mainstay of teaching surgery. Traditionally, courses and workshops are conducted in person with cadaveric specimens. This is associated with significant direct costs from the cadaveric material and indirect costs from travel and time away from one's surgical practice. Cadaveric specimens also carry an infection risk and may have undergone previous sinonasal surgery and have unpredictable sinus anatomy which can all affect the training experience. The recent COVID- 19 pandemic has had a significant global impact on the ability to conduct such courses due to its restrictions on travel, social distancing regulations, and the infection risk.

Recent advanced 3D printing techniques permit the creation of 3D sinus models based on Computed tomography (CT) scans of the paranasal sinuses. As the quality of the printing materials used, continues to improve, the tactile, “real-life” feel of the tissues make such models attractive alternatives to cadavers. Furthermore, they have the advantage of predictable anatomy and the lack of human tissue means that these courses can be outside of cadaveric facilities.

Here, we report the world-first remote FESS training course between Japan and Australia, utilizing the novel 3D sinus models in combination with telemedicine software enabling a real time supervision of step-by-step dissections. We also examined the educational effect of the remote surgical training system.

## Materials and Methods

### 3D Sinus Model

Three advanced manufactured 3D sinus models were used in the course (Fusetech, Adelaide, South Australia, Table 1). The models were printed from the axial CT scans of patients with chronic rhinosinusitis who had later undergone surgery by the senior authors. Currently, eight different models (Model 1 to Model 8) are available, all of differing degrees of complexity. Prior to the course, three of the models (Model 2, Model 6, and Model 8) were chosen by the Adelaide team based on their differing degrees of frontal sinus anatomy complexity as outlined in the international consensus papers by Wormald et al. international frontal sinus anatomy classification (1, 2). These models were printed and shipped to Japan from Australia, prior to the course. The exact same models were prepared in Adelaide for demonstration by the instructors. Because only the 3D sinus models were used in this study, ethical approval for this study was waived by the ethical board of Hokkaido University Hospital.

**Table 1:** Anatomical characteristics of the advanced manufactured 3D sinus models used in the course.

**Training round**	**Model**	**Anterior cells**	**Posterior cells**	**Medial cells**	**AP-diameter (mm)**	**Difficulty grade**	**FSDP**
Round 1	Model 2 Lt	ANC, SAFC	BE, SBC		10.9	2	AM
Round 2	Model 2 Rt	ANC,SAFC	BE, SBC		8.2	2	PL
Round 3	Model 6 Rt	ANC, SAFC	BE, SBFC		12.8	3	AM
Round 4	Model 8 Lt	ANC, SAFC	BE	FSC	6.6	4	AM
Round 5	Model 8 Rt	ANC, SAFC	BE, SBFC	FSC	7.5	4	AM

*Lt, left; Rt, right; ANC, agger nasi cell; SAFC, supra agger frontal cell; BE, bulla ethmoidalis; SBC, supra bulla cell; SBFC, supra bulla frontal cell; FSC, frontal septal cell; AP-diameter, anterior-posterior-diameter; FSDP, frontal sinus drainage pathway; AM, anterior-medial; PL, posterior-lateral*.

### Surgical Stations

Three surgical training station were prepared in the conference room of the Department of Otolaryngology-Head and Neck Surgery, Hokkaido University, Japan. Each station was equipped with endoscope and sinus surgical equipment provided by Karl Storz (Tuttlingen, Germany), a microdebrider (Medtronic, Jacksonville USA), Telepacks (Karl Storz, Tuttlingen, Germany), a Fusion image guidance system (Medtronic, Jacksonville USA), and a windows PC. The endoscope monitor was connected to the USB port of each window PC through two conversion cables (DVI to HDMI and HDMI to USB). The three endoscopic images during dissection were simultaneously shared with 2 rhinologists based in Adelaide, Australia, on an encrypted telemedicine platform, Quintree (Michigan, USA).

### Surgical Training

Prior to dissecting three Japanese surgeons were trained by the Adelaide Rhinologists AJP and PJW to assess the frontal recess anatomy and evaluate the FDSP using the computerized Scopis “Building Block Software” (Stryker, Michigan, USA). The Japanese surgeons were then asked to plan and conduct the frontal sinus dissection for the left side of Model 2, beginning with uncinectomy, followed by resections of the anterior wall of ANC, identification of FSDP, and resection of all cells in the frontal recess. Sharing the three endoscopic views, the instructors in Adelaide directly supervised the dissectors in Japan performing the surgical procedures and gave them real-time feedback and instruction using the Quintree platform. Following completion of their first dissection, the participating surgeons watched the instructors perform the dissection on the exact same 3D sinus model (the left side of Model 2) in Australia. At the end of the model dissection a video-recording of the actual real-life surgery of the patient from whom the model had been printed was shown. This process was repeated for each of the remaining four dissections with increasing frontal recess complexity on each dissection (right side of Model 2, right side of Model 6, and left and right sides of Model 8, Table 1). All the dissectors conducted the dissection of the same models. As a next step the endoscopic modified Lothrop procedure (EMLP, frontal drillout) was conducted by the participating surgeons using model 2, following the demonstration of the procedure by the instructors. The course was broadcasted worldwide to over 200 otolaryngologists' personal computers using the web-conferencing platform Zoom (California, USA). Social distancing of all involved was possible and enforced.

### The Time to Identify FSDP

During the course, the time to identify the FSDP was measured. It was defined as the time interval from starting the resection of the anterior wall of ANC until the insertion of the tip of a malleable sinus probe or suction curette into the FSDP. The time was standardized as a relative value to the time taken for the instructors in Adelaide to perform the same dissection.

### Measurement of Volume of FSDP in the Models Before and After the Training

CT examinations of the 3D sinus models before and after the training were performed using a 16-slice multidetector-row CT scanner (Hitachi, Tokyo, Japan) with collimation of 0.63 mm at 120 kV and 200 mA or less and a rotation time of 1.0 s. Coronal and sagittal multiplanar reconstruction (MPR) images were obtained from the axial images. 3D computed graphic images of FSPD were created, and the volume of FSDP from the level of the superior edge of 3D sinus models to the level of the floor of ANC was quantified by 3D image analysis system, SYNAPSE VINCENT (Fujifilm, Tokyo, Japan, Supplementary Figure 1). For model 2 (EMLP plus bilateral FSDP), the total volume of bilateral FSDP was calculated and compared before and after the surgeries. For model 6 and 8 frontal sinusotomy without frontal drillout was performed and the volume of each side of FSDP were compared before and after the frontal sinusotomy.

### Questionnaires for the Trainers and Audiences

During the course, subjective data was collected from the three dissectors using a visual-analog (VAS) scale. The data included (1) subjective difficulty of the surgeries during the frontal sinusotomy, (2) subjective completeness of frontal sinusotomy, and (3) confidence in performing a frontal sinusotomy in general.

After the course, a questionnaire survey was performed on dissectors and audiences, to evaluate the usefulness of instruction, the quality of the telemedicine platform, and the educational value. Each response was converted to the VAS score (0–100).

### Statistical Analysis

All data is expressed as the mean ± Standard Deviation (SD). The continuous variables were compared with a two-tailed Student's *t*-test. *P*-values of < 0.05 were considered statistically significant. All analyses were performed using the JMP® 11 (SAS Institute Inc., Cary, NC, USA).

## Results

### Course and Dissectors

**Figure 1:**
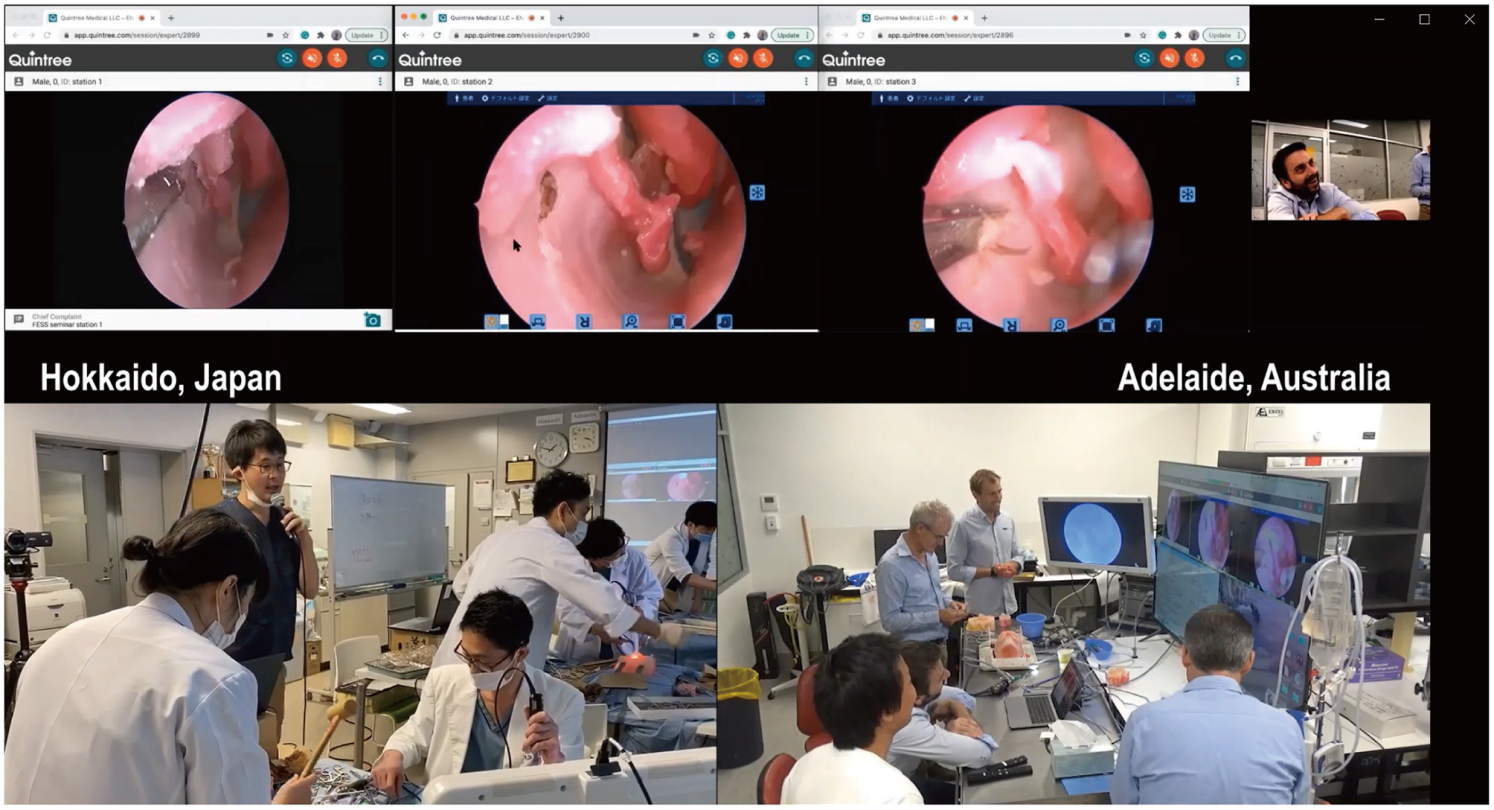
The remote endoscopic surgery training course was conducted between Hokkaido, Japan, and Adelaide, Australia, and broadcasted worldwide. The course was held over 2 days in February 2021. Three otolaryngologists in Hokkaido were trained to perform frontal sinus dissections on 3D sinus models printed from CT scans of patients. Two rhinologists based in Adelaide, 8,000-km away from Hokkaido, simultaneously viewed the surgeries and provided real-time feedback using a telemedicine platform, Quintree. The course was also broadcast worldwide to over 200 otolaryngologists' personal computers.

The course was held over 2 days in February 2021 in the conference room of the Department of Otolaryngology-Head and Neck Surgery, Hokkaido University, in Japan. The experience years of the Japanese Otolaryngologists was 9.7 ± 7.6 years. Two were officially certified board members of the Japanese Otolaryngology Society, and the other was an otolaryngology registrar yet certified. None of them were trained rhinologists. Their operative experience in performing FESS and frontal sinusotomies was 101.7 ± 60.1 and 21.7 ± 18.9 sides, respectively. The registrar had no experience in performing frontal sinusotomies before the course. None of the three dissectors had performed a frontal drillout before. All dissectors performed five frontal sinusotomies and one frontal drillout under the supervision and real-time feedback by the instructors in Adelaide (Figure 1 and Supplementary Video 1).

### The Remote Endoscopic Surgery Training Course Was Objectively and Subjectively Beneficial in Improving the Dissectors' Skills

**Figure 2:**
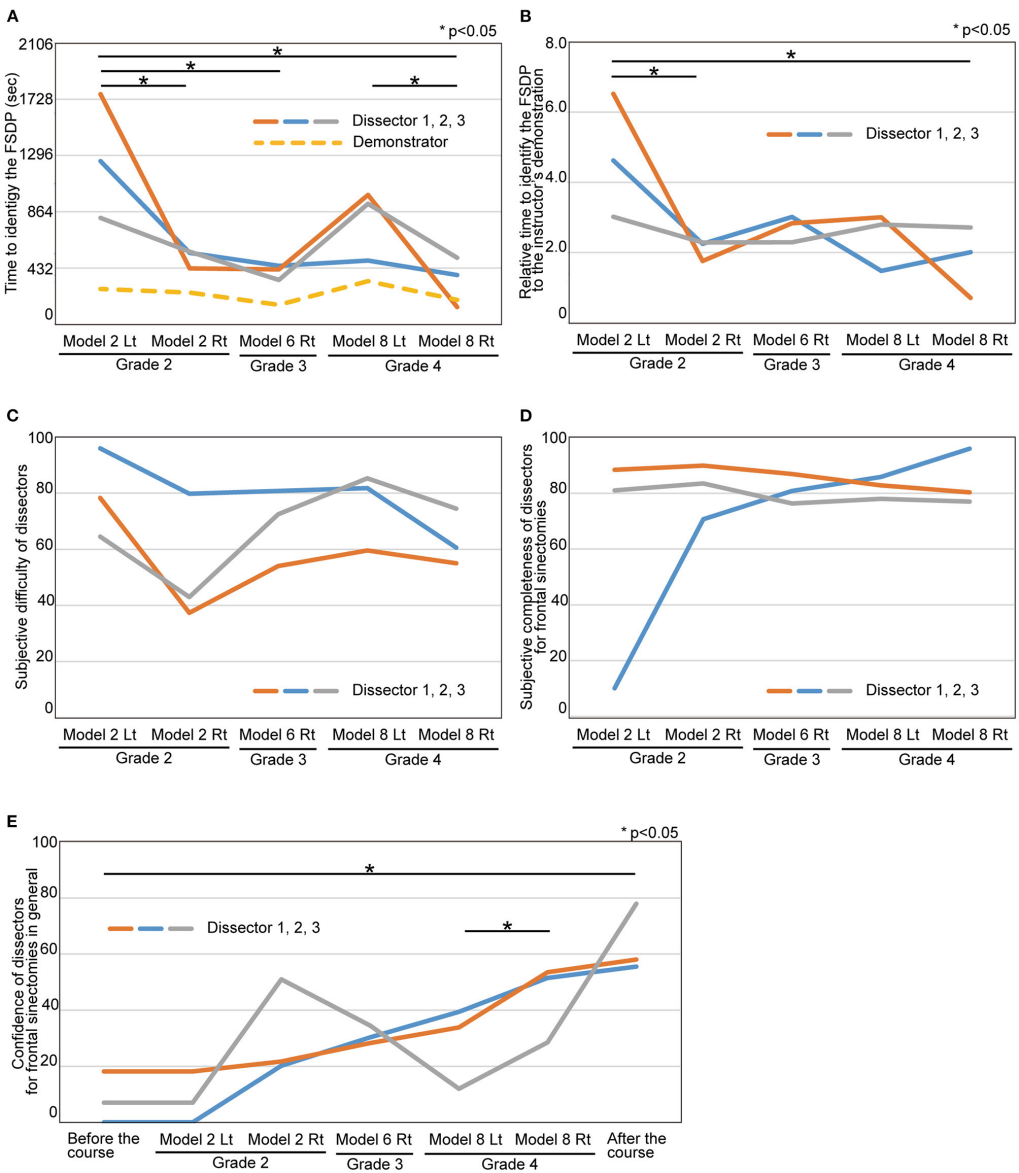
The remote endoscopic surgery training course was objectively and subjectively beneficial in improving the dissectors' surgical techniques. **(A)** The time to identify the FSDP. It was defined as the time interval from starting to resect the anterior wall of ANC to identify and insert a ball probe tip to FSDP at the height of the top of ANC. *P*-values for indicated comparisons were determined by *t*-test. *p < 0.05. **(B)** The relative time to identify the FSDP to the instructor's demonstration. *P*-values for indicated comparisons were determined by *t*-test. *p < 0.05. **(C)** The subjective difficulty of the surgeries that the dissectors were perceiving during the dissection. **(D)** The subjective completeness of frontal sinusotomy during the dissection. **(E)** The confidence for frontal sinusotomy in general before, during, and after the course. *P*-values for indicated comparisons were determined by *t*-test. *p < 0.05.

To evaluate the educational impact of the course, we examined how long it took for the dissectors to identify the FSDP. The duration from starting to resect the anterior wall of ANC to identify and insert a malleable probe or frontal sinus curette into the FSDP was measured. In the first round, the dissectors needed 1,292 ± 672 s to identify the FSDP. As the course proceeded, the time to identify FSDP decreased and to 321 ± 267 s at the final side despite the most complexity anatomy (Figure 2A, *p* = 0.017). The time was also expressed as relative time to the instructor's demonstration. The relative time also has significantly shortened (from 4.77 ± 2.48-fold at the first round to 1.71 ± 1.42-fold, *p* = 0.034, Figure 2B), even though the difficulty of anatomies has increased (Figure 2).

**Figure 3:**
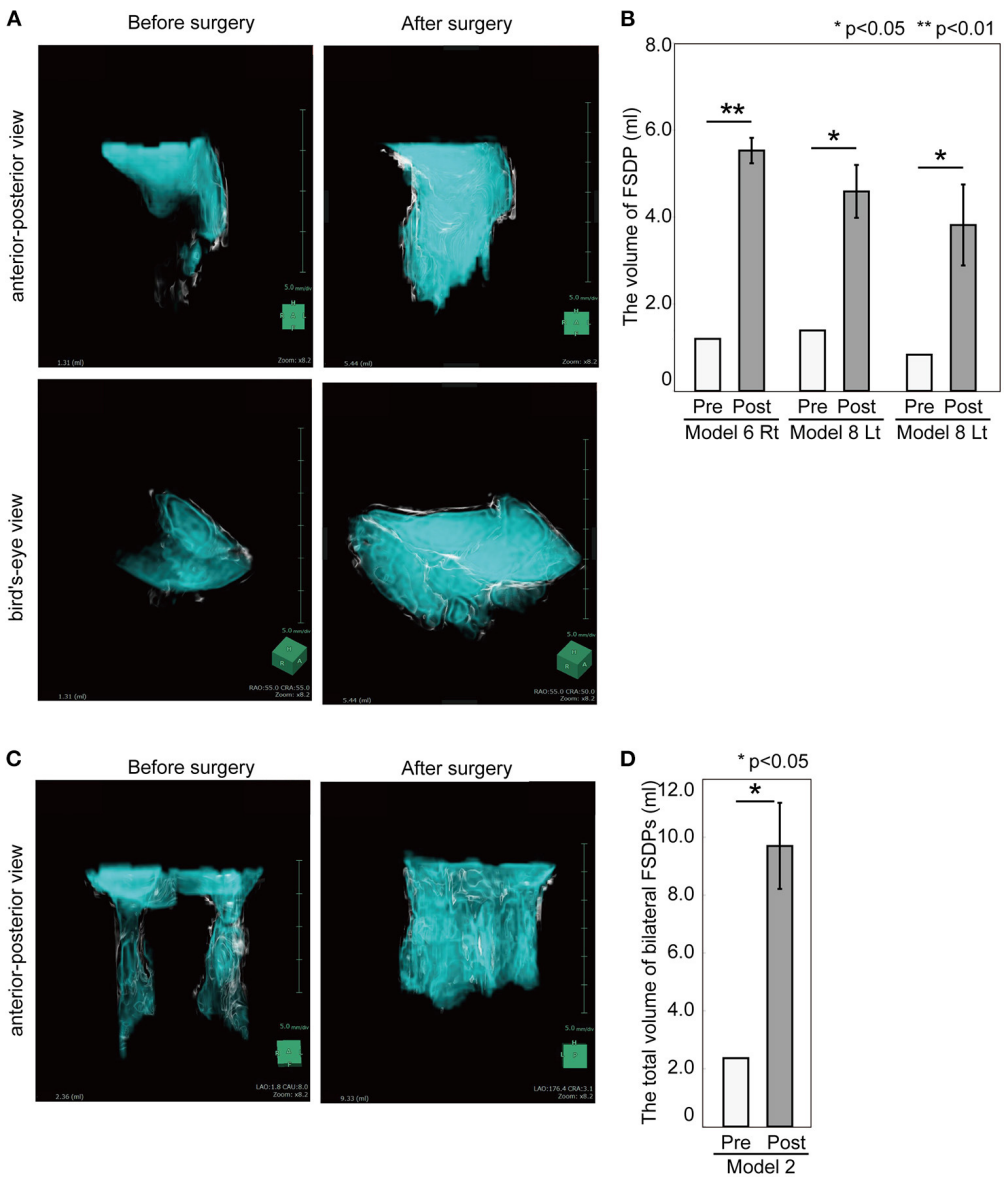
The volume of FSDP before and after the frontal sinusotomies and the frontal drill out. **(A)** 3D computed graphic images of right FSDP in the model 6 before and after the sinusotomies. **(B)** The volume of FSDP in 3D sinus models was significantly improved after the frontal sinusotomies than before the procedure. *P*-values for indicated comparisons were determined by *t*-test. *p < 0.05, **p < 0.01. **(C)** 3D computed graphic images of bilateral FSDP in the 3D sinus models before and after the frontal drillout (the model 2). **(D)** The total volume of bilateral FSDPs was also significantly improved after the frontal drillout. *P*-values for indicated comparisons were determined by *t*-test. *p < 0.05.

The CT images of 3D sinus models before and after the training were taken and the volume of FSDP was calculated and compared. Image analysis showed the volume of FSDP increased significantly after the frontal sinusotomy (Model 6 Rt; 1.20 ± 0.00 ml vs. 5.53 ± 0.29 ml, *p* = 0.002, Model 8 Lt; 1.39 ± 0.00 ml vs. 4.59 ± 0.93 ml, *p* = 0.027, and Model 8 Rt; 0.83 ± 0.00 ml vs. 3.82 ± 0.61 ml, *p* = 0.014, Figures 3A,B). The total volume of bilateral FSDP after frontal drillout was significantly improved from 2.36 ± 0.00 to 9.70 ± 1.49 ml postoperatively (Model 2, *p* = 0.014, Figures 3C,D).

The subjective difficulty of surgeries perceived by the dissectors during the dissections did not significantly change throughout the course (the first dissection; 78.60 ± 20.45, the last dissection; 65.91 ± 9.47, *p* = 0.53, Figure 2C) despite an increase in the difficulty of preoperative comprehension of the anatomies (Supplementary Figure 2). The subjective completeness of frontal sinusotomy was quickly increased and reached the plateau (Figure 2D). The confidence to perform a frontal sinusotomy in significantly increased throughout the course (before the course; 8.39 ± 9.17, after the course; 63.88 ± 12.29, *p* = 0.025, Figure 2E).

### The Remote Endoscopic Surgery Training Course Is Beneficial Not Only for Dissectors but Also for Audiences Away From the Training Room

**Figure 4:**
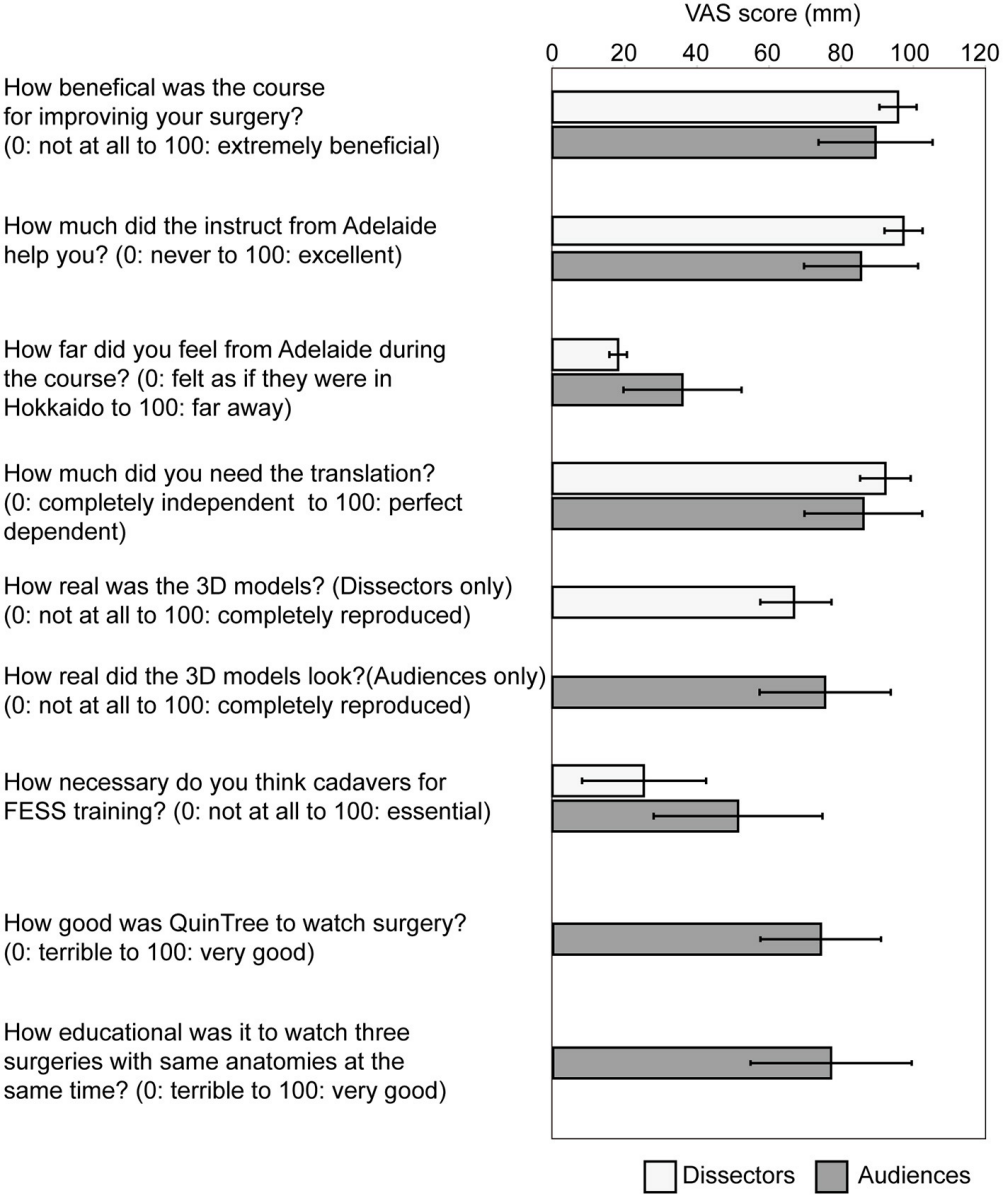
The result of questionnaire from the dissectors and the audiences on educational benefits of the remote FESS training course. A questionnaire survey was performed on dissectors and audiences, including the following topic; the general benefit of the course to improve the surgical skills (0: not at all to 100: extremely beneficial), the usefulness of instructions from Adelaide (0: not at all to 100: excellent), subjective distance from Adelaide (0: felt as if they were in Hokkaido to 100: far away), the dependency on the translation between English and Japanese (0: completely independent to 100: perfect dependent), the reproducibility of the 3D sinus models to the real nasal cavity and paranasal sinuses (0: not at all to 100: completely reproduced, for dissectors only), the reproducibility of the 3D sinus models to the real nasal cavity and paranasal sinuses when watched on monitors (0: not at all to 100: completely reproduced, for audiences only), the necessity of using cadavers for the similar surgical training (0: not at all to 100: essential), the quality of the communication by Quintree (0: terrible to 100: very good), and the educational effect of watching the three surgeries with same anatomies at the same time by Quintree (0: terrible to 100: very good). Data were gathered by visual-analog scale and expressed as the mean ± SD.

The questionnaires were answered by the three dissectors (100%) and 79 of the 200 audience members (38.73%). The course's perceived general benefit to improve surgical skills was 95.47 ± 5.13 in the dissectors and 89.24 ± 15.75 in the audience (0: not at all to 100: extremely beneficial). The usefulness of instruction provided by the Adelaide supervisors was 96.97 ± 5.25 in the dissectors and 82.26 ± 15.73 in the audience (0: never to 100: excellent). Subjective distance from Adelaide (How far they felt from the Adelaide team) was 18.13 ± 2.40 in the dissectors and 35.90 ± 16.31 in the audience, respectively (0: felt as if the instructors were in the training room in Hokkaido to 100: far away). The dependency on the language translation provided by the first author for those who couldn't understand English was 91.96 ± 2.40 in the dissector and 85.90 ± 16.31 in the audience (0: completely independent to 100: perfect dependent) (Figure 4).

We also evaluated how the dissectors perceived the models. The surgical likeness of the model to the tissue feel anatomy of a real sinonasal cavity was 66.72 ± 15.04 in the dissectors (0: not at all to 100: completely reproduced). Similarly, the audience was asked how realistic the anatomy of the 3D sinus models looked compared to typical endoscopic sinonasal anatomy that they are accustomed to seeing in their practice. The reproduction of the 3D sinus model watched on the monitor was 75.32 ± 18.12 in the audiences (0: not at all to 100: completely reproduced). Obviously the tissue feel of the 3D sinus models could not be commented on by the non-dissecting audience members.

The perceived necessity of needing to use cadavers for similar surgical training after participating in this course was 25.24 ± 19.21 in the dissectors and 51.28 ± 23.31 in the audiences (0: not at all to 100: absolutely necessary) (Figure 4).

The quality of the telemedicine platform provided by Quintree was as high as 73.42 ± 16.57 (0: terrible to 100: very good). The educational value to the non-dissecting audience of simultaneously watching the three surgeons performing the same surgery on three identical frontal sinus models was 76.27 ± 22.11 (0: terrible to 100: very good) with 84.81% of the audience considering the educational effect of the three simultaneous dissections as higher compared to watch only one dissection (Figure 4).

## Discussion

This study documents, for the first time, the feasibility of performing a remote endoscopic sinus surgical course using non-cadaveric advanced material 3D sinus models in conjunction with an encrypted telemedicine platform. Post-course evaluation demonstrated that in addition to such a course being possible, it objectively and subjectively improved surgeon's frontal sinus surgical skill in terms of efficiency and completeness of dissection. Participants and observers described a degree of post-course satisfaction in terms of learning opportunity.

With advanced in 3D printing technology and manufacturing many 3D organ models have been developed for surgical training, including temporal bone (6–8), paranasal sinuses (9–12), skull base (9, 13–16), kidney, renal pelvis, and ureter (17), mandibular (9), aorta (18), and heart (19). In addition, some models successfully reproduce the organs with pre-existing pathology such as cerebral aneurysms (20, 21) and basilar invagination (22). Studies assessing the use of 3D printed models for surgical training have generally supported their benefits for surgical education (23) and recently in the field of veterinary medicine, Kelly reported the use of 3D models to teach students surgical anatomy remotely (24). Several additional small studies assessing remote training for surgical skills have also been reported. These generally involve the teaching of basic surgical skills using box trainers such as peg transfer, knot tying, and suturing techniques under laparoscopy (25, 26). Recently a course utilizing remote guidance of actual endovascular surgery using augmented reality (AR), has also been published (27). To our knowledge, our study is the first remote course to assess the utility of using 3D-printed models for a complex surgical procedure in human based models.

3D sinus models hold several advantages over cadavers in surgical training. There is no risk of infectivity from the specimens, the courses can be held outside of designated anatomical institutions required for housing cadavers, and there is an elimination of the ethical issues that would accompany the transmission of cadaveric pictures on a telemedicine platform. Furthermore, such models are much cheaper than cadavers, ~1/10 of the cost, meaning that course costs can be kept lower, and candidates can have the opportunity to dissect more frontal sinuses during a course than they have historically been able to do with cadaver.

Another major advantage of using advance material 3D sinus models over cadavers is the ability to print standardized anatomy, that allows both instructors and students to teach and learn in a predictable and graduated fashion as we saw in this course. From an instructor's perspective, pre-existing knowledge of the anatomy is likely to translate into more effective and efficient teaching. From a student's perspective, the student can gain knowledge, familiarity and confidence from performing more simple frontal sinus dissections before attempting more difficult ones. Furthermore, they can also learn from watching surgery performed by the expert's on exactly the same anatomy.

The successful completion of this course by all three surgeons validates the initial objective of this study, which was to see if it was possible to use encrypted telemedicine software to remotely teach and observe sinus surgery in a structured environment. Furthermore, it confirmed that surgery as complex as frontal recess dissection, could be taught in a step-wise and structured manner, using the building block concept, to surgeons of differing levels of sinus surgical experience, including a trainee that had never performed a sinus procedure prior. Of even greater significance, and further supporting this way of teaching, was the objective evidence that despite an increase in the difficulty of frontal sinus anatomy, surgical time and complete of dissection both improved as the course went on.

The results from the feedback questionnaire obtained from both the dissectors and the general audience confirmed the high level of satisfaction the participants obtained from attending the course. In terms of the specific questions of the questionnaire, the physical distance between the delegates and instructors did not pose a problem due to the clear instruction of the instructors facilitated by the innovative telemedicine technology utilized. There was a general consensus that the 3D sinus models were a suitable alternative to the cadaveric models particularly from the dissectors who were able to directly work with the model. A distinct advantage of the 3D sinus model as outlined by the responses of both the dissectors and the general audience, was the predictability and reproducibility of the model when compared to cadaveric specimens. The importance of well-performed translation was identified by over half of the Japanese audience as a significant factor contributing to their satisfaction with the course. This is obviously of critical importance when the native language of the delegates is not that of the remote instructors.

In addition to the high level of delegate and audience satisfaction from the course, both instructors (AJP and PJW) also reported high level of satisfaction with the ease of which they were able to teach (data not shown). Using the Quintree telemedicine platform they were able to visualize the endoscopic images of all three candidates on the same screen simultaneously, and so were in direct contact with each of the candidates for their duration of their dissection. This allowed them to tailor the extent and level of their instruction to the need of the candidate. This is not possible with one-on-one supervision required during face-face in person courses.

The obvious limitations of this study include the small number of dissecting surgeons assessed and the varying surgical experience of the dissectors and the audience. We recognize that this has the protentional to create bias in some of the responses evaluating the utility of the course. With this said, the primary aim of the course was to assess whether we could address the issues of conducting educational surgical courses, posed by the COVID-19 pandemic. With the promising results obtained from the feedback from this course, we plan to conduct a much larger course with more surgical dissectors whereby factors such as the relationship of surgical experience to learning benefit from such courses can be more adequately assessed.

## Conclusion

To the best of our knowledge, this is the first study to report on a remotely conducted FESS training course using advanced 3D sinus models with different grades of complexity. This study demonstrated that remote courses and the use of non-cadaveric material may provide a suitable alternative to the traditional cadaveric/in person courses. This is particularly relevant in the current environment of the COVID-19 pandemic.

## Data Availability Statement

The raw data supporting the conclusions of this article will be made available by the authors, without undue reservation.

## Ethics Statement

Ethical review and approval was not required for the study on human participants in accordance with the local legislation and institutional requirements. The patients/participants provided their written informed consent to participate in this study. Written informed consent was obtained from the individual(s) for the publication of any potentially identifiable images or data included in this article.

## Author Contributions

MS, EV, and PW designed the project. AH and AP supervised the project. MS, EV, and KO set up the course. PW and AP supervised the training. KM and YN analyzed CT images. MS, EV, and AP wrote the draft. All authors provided feedback on the manuscript.

## Funding

This study was supported by JSPS KAKENHI Grant Number 18KK0444, Itoiyaku Gakuzyutsu Zaidan, and Suginome Kinen Kai for MS.

## Conflict of Interest

MS: receiving royalties from Igakushoin. PW: consultant for Fusetec, Neilmed and receiving royalties from Medtronic, Fusetec, and Integra. Shareholder for Chitogel. AP: consultant for Fusetec, Medtronic, Tissium, and ENT technologies. Shareholder for Chitogel and Speakers Bureau for Sequiris. The remaining authors declare that the research was conducted in the absence of any commercial or financial relationships that could be construed as a potential conflict of interest.

## Publisher's Note

All claims expressed in this article are solely those of the authors and do not necessarily represent those of their affiliated organizations, or those of the publisher, the editors and the reviewers. Any product that may be evaluated in this article, or claim that may be made by its manufacturer, is not guaranteed or endorsed by the publisher.

## Supplementary Material

Supplementary Figure 1 Measurement of the volume of the frontal sinus drainage pathway. **(A)** 3D sinus models used in the course. The anatomy is identical to that of humans as they are printed from the axial CT scans of patients with chronic rhinosinusitis. The materials mimic human tissue found in the nasal cavity and sinuses. **(B–D)** CT images of the 3D sinus models were acquired before and after the dissection. 3D computed graphic images of FSPD (sky blue) were created by a 3D image analysis system, SYNAPSE VINCENT with different transparency of the 3D sinus models from 0% **(B)** to 100% **(D)**. The volume of FSDP from the level of the superior edge of 3D sinus models to the level of the floor of ANC was quantified.

Supplementary Figure 2 Subjective difficulty of preoperative comprehension of anatomies of frontal recess in each difficulty grade. The subjective difficulty of prediction of FSDP with the Building Block Concept was increased with the higher difficulty grade. *P*-values for indicated comparisons were determined by *t*-test. ^*^*p* < 0.05, ^**^*p* < 0.01.

## References

[1] Fokkens WJ Lund VJ Hopkins C Hellings PW Kern R Reitsma S. European position paper on rhinosinusitis and nasal polyps 2020. Rhinology. (2020) 58(Suppl.S29):1–464. 10.4193/Rhin20.601 32077450

[2] Wormald P-J. Endoscopic Sinus Surgery. Stuttgart, New York, NY: Thieme (2018). 10.1055/b-0038-149997

[3] Naidoo Y Wen D Bassiouni A Keen M Wormald PJ. Long-Term results after primary frontal sinus surgery. Int Forum Allergy Rhinol. (2012) 2:185–90. 10.1002/alr.21015 22253224

[4] Wormald PJ. Surgery of the frontal recess and frontal sinus. Rhinology. (2005) 43:82–5. 16008060

[5] Wormald PJ. The agger nasi cell: the key to understanding the anatomy of the frontal recess. Otolaryngol Head Neck Surg. (2003) 129:497–507. 10.1016/S0194-5998(03)01581-X 14595272

[6] Chien WW da Cruz MJ Francis HW. Validation of a 3d-Printed human temporal bone model for otology surgical skill training. World J Otorhinolaryngol Head Neck Surg. (2021) 7:88–93. 10.1016/j.wjorl.2020.12.004 33997717 PMC8103535

[7] Da Cruz MJ Francis HW. Face and content validation of a novel three-dimensional printed temporal bone for surgical skills development. J Laryngol Otol. (2015) 129(Suppl.3):S23–9. 10.1017/S0022215115001346 26073332

[8] Mooney MA Cavallo C Zhou JJ Bohl MA Belykh E Gandhi S. Three-dimensional printed models for lateral skull base surgical training: anatomy and simulation of the transtemporal approaches. Oper Neurosurg. (2020) 18:193–201. 10.1093/ons/opz120 31172189

[9] Chan HH Siewerdsen JH Vescan A Daly MJ Prisman E Irish JC. 3D rapid prototyping for otolaryngology-head and neck surgery: applications in image-guidance, surgical simulation and patient-specific modeling. PLoS ONE. (2015) 10:e0136370. 10.1371/journal.pone.0136370 26331717 PMC4557980

[10] Chang DR Lin RP Bowe S Bunegin L Weitzel EK McMains KC. Fabrication and validation of a low-cost, medium-fidelity silicone injection molded endoscopic sinus surgery simulation model. Laryngoscope. (2017) 127:781–6. 10.1002/lary.26370 28000224

[11] Alrasheed AS Nguyen LHP Mongeau L Funnell WRJ Tewfik MA. Development and validation of a 3d-printed model of the ostiomeatal complex and frontal sinus for endoscopic sinus surgery training. Int Forum Allergy Rhinol. (2017) 7:837–41. 10.1002/alr.21960 28614638

[12] Barber SR Jain S Son YJ Chang EH. Virtual functional endoscopic sinus surgery simulation with 3d-printed models for mixed-reality nasal endoscopy. Otolaryngol Head Neck Surg. (2018) 159:933–7. 10.1177/0194599818797586 30200812

[13] Tai BL Wang AC Joseph JR Wang PI Sullivan SE McKean EL. A physical simulator for endoscopic endonasal drilling techniques: technical note. J Neurosurg. (2016) 124:811–6. 10.3171/2015.3.JNS1552 26339850

[14] Hsieh TY Cervenka B Dedhia R Strong EB Steele T. Assessment of a patient-specific, 3-dimensionally printed endoscopic sinus and skull base surgical model. J Am Med Assoc Otolaryngol Head Neck Surg. (2018) 144:574–9. 10.1001/jamaoto.2018.0473 29799965 PMC6145784

[15] Zheng JP Li CZ Chen GQ Song GD Zhang YZ. Three-Dimensional printed skull base simulation for transnasal endoscopic surgical training. World Neurosurg. (2018) 111:e773–82. 10.1016/j.wneu.2017.12.169 29309974

[16] Ding CY Yi XH Jiang CZ Xu H Yan XR Zhang YL. Development and validation of a multi-color model using 3-Dimensional printing technology for endoscopic endonasal surgical training. Am J Transl Res. (2019) 11:1040–8. 10.3171/2019.6.FOCUS19294 30899403 PMC6413258

[17] Cheung CL Looi T Lendvay TS Drake JM Farhat WA. Use of 3-Dimensional printing technology and silicone modeling in surgical simulation: development and face validation in pediatric laparoscopic pyeloplasty. J Surg Educ. (2014) 71:762–7. 10.1016/j.jsurg.2014.03.001 24776857

[18] Hussein N Honjo O Barron DJ Yoo SJ. Supravalvular aortic stenosis repair: surgical training of 2 repair techniques using 3d-printed models. Interact Cardiovasc Thorac Surg. (2021) 2021:ivab198. 10.1093/icvts/ivab198 34378022 PMC8669556

[19] Yildiz O Köse B Tanidir IC Pekkan K Güzeltaş A Haydin S. Single-center experience with routine clinical use of 3d technologies in surgical planning for pediatric patients with complex congenital heart disease. Diagn Interv Radiol. (2021) 27:488–96. 10.5152/dir.2021.20163 34313233 PMC8289433

[20] Mashiko T Kaneko N Konno T Otani K Nagayama R Watanabe E. Training in cerebral aneurysm clipping using self-made 3-dimensional models. J Surg Educ. (2017) 74:681–9. 10.1016/j.jsurg.2016.12.010 28110854

[21] Nagassa RG McMenamin PG Adams JW Quayle MR Rosenfeld JV. Advanced 3d printed model of middle cerebral artery aneurysms for neurosurgery simulation. 3D Print Med. (2019) 5:11. 10.1186/s41205-019-0048-9 31372773 PMC6743137

[22] Narayanan V Narayanan P Rajagopalan R Karuppiah R Rahman ZA Wormald PJ. Endoscopic skull base training using 3d printed models with pre-existing pathology. Eur Arch Otorhinolaryngol. (2015) 272:753–7. 10.1007/s00405-014-3300-3 25294050

[23] Langridge B Momin S Coumbe B Woin E Griffin M Butler P. Systematic review of the use of 3-dimensional printing in surgical teaching and assessment. J Surg Educ. (2018) 75:209–21. 10.1016/j.jsurg.2017.06.033 28729190

[24] Thieman Mankin KM Cornell K Peycke L Dickerson V Scallan E. Adaptation of a hands-on veterinary surgical training course from a traditionally taught laboratory to a remotely taught laboratory during a global pandemic. Vet Surg. (2021) 50:494–506. 10.1111/vsu.13584 33565116

[25] Quaranto BR Lamb M Traversone J Hu J Lukan J Cooper C. Development of an interactive remote basic surgical skills mini-curriculum for medical students during the covid-19 pandemic. Surg Innov. (2021) 28:220–5. 10.1177/15533506211003548 33780641

[26] Sloth SB Jensen RD Seyer-Hansen M Christensen MK De Win G. Remote training in laparoscopy: a randomized trial comparing home-based self-regulated training to centralized instructor-regulated training. Surg Endosc. (2021) 7:1–12. 10.1007/s00464-021-08429-7 33742271 PMC7978167

[27] Hassan AE Desai SK Georgiadis AL Tekle WG. Augmented reality enhanced tele-proctoring platform to intraoperatively support a neuro-endovascular surgery fellow. Interv Neuroradiol. (2021). 10.1177/15910199211035304 PMC9185096 34346826

