# Supplementary figures and images for: Remote Training of Functional Endoscopic Sinus Surgery With Advanced Manufactured 3D Sinus Models and a Telemedicine System
Source: Front Surg. 2021 Oct 1;8:746837. doi: 10.3389/fsurg.2021.746837 (PMC8517106; doi:10.3389/fsurg.2021.746837)

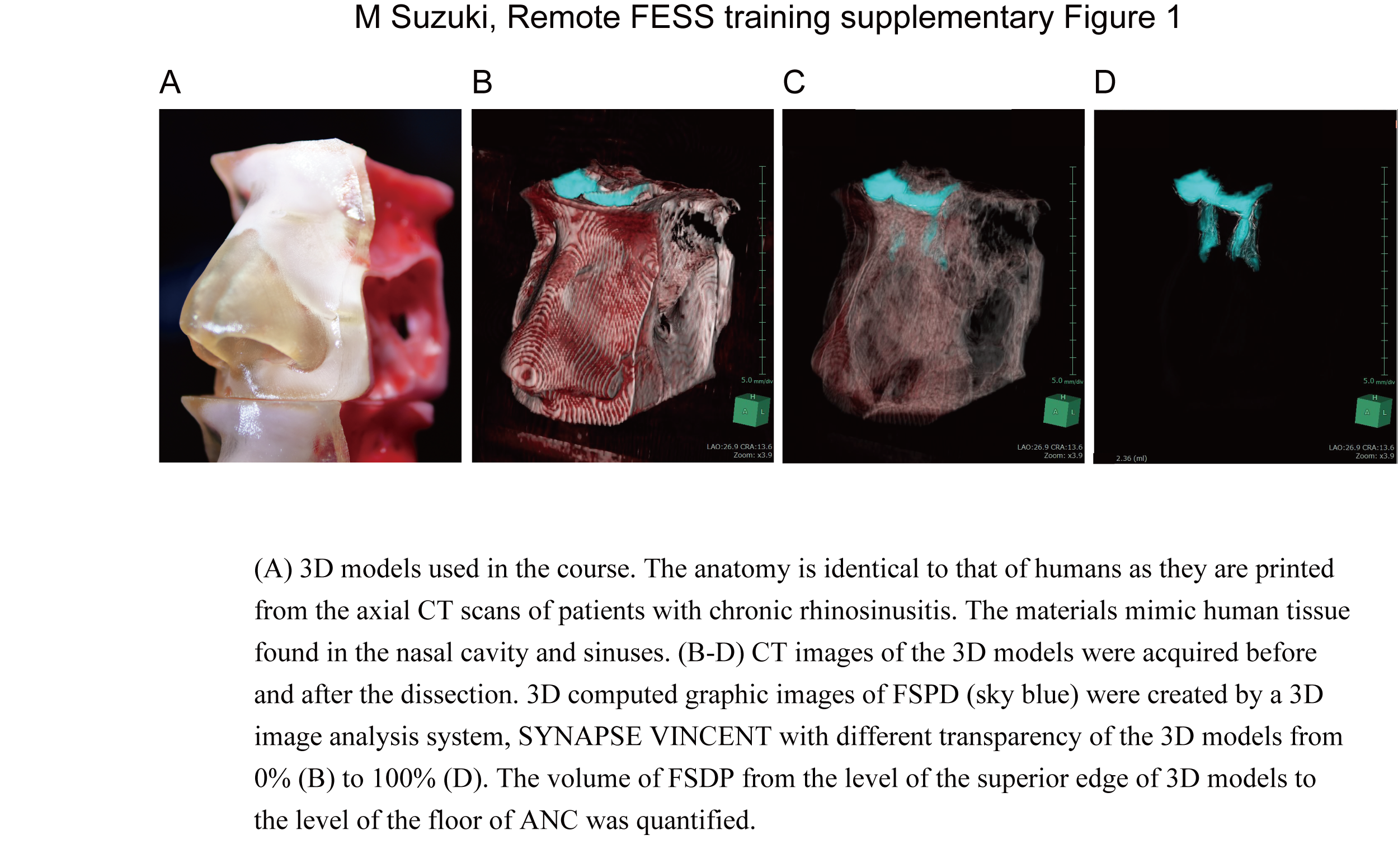

Supplement: Supplementary Figure 1 — Measurement of the volume of the frontal sinus drainage pathway. (A) 3D sinus models used in the course. The anatomy is identical to that of humans as they are printed from the axial CT scans of patients with chronic rhinosinusitis. The materials mimic human tissue found in the nasal cavity and sinuses. (B–D) CT images of the 3D sinus models were acquired before and after the dissection. 3D computed graphic images of FSPD (sky blue) were created by a 3D image analysis system, SYNAPSE VINCENT with different transparency of the 3D sinus models from 0% (B) to 100% (D). The volume of FSDP from the level of the superior edge of 3D sinus models to the level of the floor of ANC was quantified. [file Image_1.TIF]

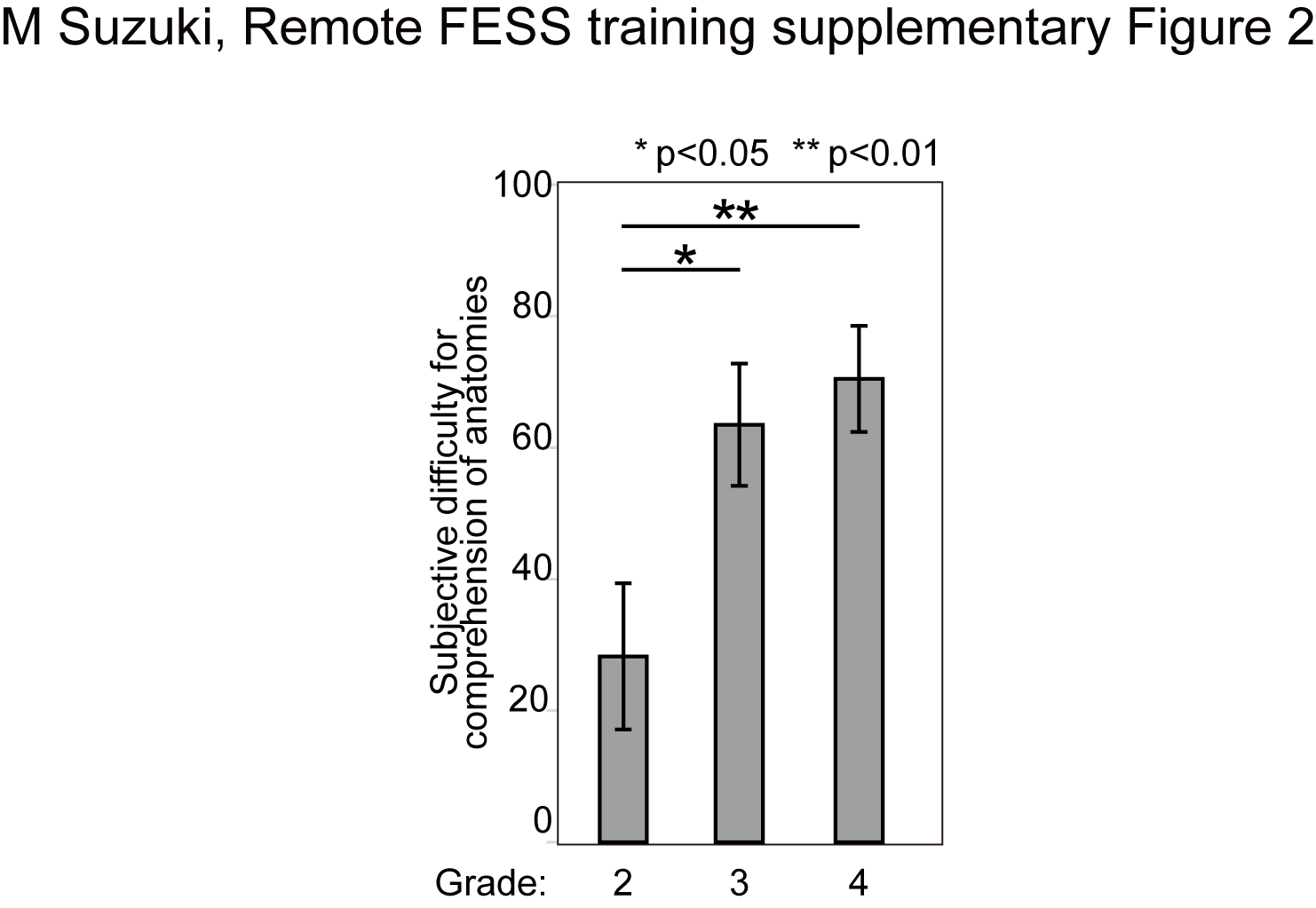

Supplement: Supplementary Figure 2 — Subjective difficulty of preoperative comprehension of anatomies of frontal recess in each difficulty grade. The subjective difficulty of prediction of FSDP with the Building Block Concept was increased with the higher difficulty grade. P-values for indicated comparisons were determined by t-test. *p < 0.05, **p < 0.01. [file Image_2.TIF]
